# Spontaneous coronary artery dissection and healing documented by optical coherence tomography

**DOI:** 10.1590/S1679-45082016AI3551

**Published:** 2016

**Authors:** Jamil Cade, Gary S Mintz, Roderick M Silva, Adriano Caixeta

**Affiliations:** 1 Hospital Israelita Albert Einstein, São Paulo, SP, Brazil; 2 Cardiovascular Research Foundation, New York, NY, USA

An otherwise healthy 57-year-old female patient with no risk factors for coronary artery disease presented to the emergency room with acute chest pain. The patient had been taking appetite suppressant (dimethylamylamine, Oxyelite Pro, (USP Labs) for the last 7 days.^(1,2)^

**Figure 1:**
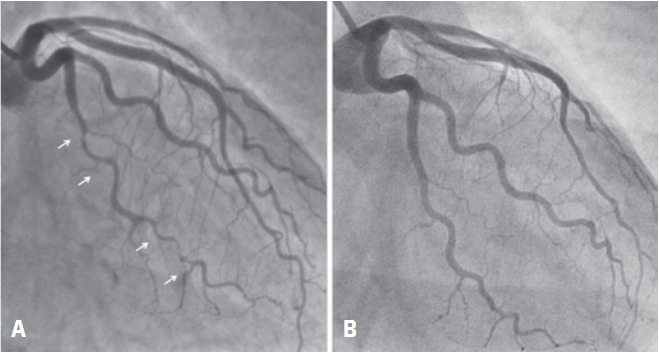
Coronary angiography (A) during hospitalization and (B) 6 months

**Figure 2:**
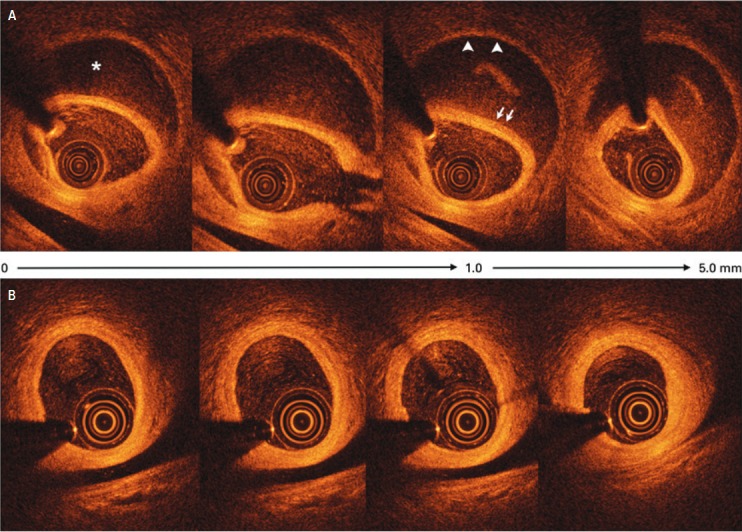
Optical coherence tomography from proximal (0mm) to distal (5mm) in the OM at (A) baseline and (B) at 6-month follow-up; the images represent the exact same locations in the artery with the spontaneous coronary dissection (A) and healing (B). White arrows indicate the intima/media and arrowheads the adventicia. Images at baseline showing intramural hematoma, intimal tear at 4 o'clock, and double lumen morphology with intimomedial dissection separating the true lumen (where probe is located) from the false lumen (asterix). Images (B) at 6-month showed a complete resolution of the hematoma and dissection. Note a 3-layer aspect with small intimal thickness

Electrocardiography showed no abnormalities although both serum creatinine kinase-MB (3.59ng/mL) and troponin I (8,310pg/mL) were elevated. Coronary angiography revealed extensive and abrupt lumen narrowing in the obtuse marginal with a subtle intraluminal defect within the distal part of the vessel (Figure 1). Optical coherence tomography (OCT), which is a novel near-infrared light based intravascular imaging modality with high-resolution images (10 to 20μm), showed an intramural hematoma that was 20mm long, with a 10mm length of near-circumferential dissection (double lumen) (Figure 2) with no evidence of atherosclerosis. The patient was discharged after medical management with aspirin, clopidogrel, low-molecular weight heparin. Six months later a new coronary angiography and OCT were performed revealing a complete spontaneous resolution of the dissection (Figures 1 and 2).

Spontaneous coronary artery dissection is an unusual, underdiagnosed disease, and its clinical presentation ranges from unstable angina to sudden cardiac death. This condition predominantly affects young women without classical cardiovascular risk factors, and it is increasingly diagnosed in those who are not in the peripartum period.^(1)^ The etiology and pathogenesis of spontaneous coronary artery dissection are not completely understood, but primary disruption with bleeding of *vasa vasorum* and intramedial hemorrhage have been proposed as the underlying mechanisms. Alternatively, an intimal tear may result in separation of coronary wall layers with the creation of a false lumen. Pressure-driven expansion of this lumen induces axial propagation of the dissection and true lumen compression, causing myocardial ischemia. Eventually, as occurred in the current case, angiography shows a long eccentric narrowing without the presence of a visible intimal flap.^(1)^

Thus, an invasive imaging modality, such as OCT or intravascular ultrasound, should be the gold standard to diagnose spontaneous coronary artery dissection,^(2)^ specially in doubtful cases by angiography. Optimal treatment is still controversial and includes medical management for asymptomatic patients with normal coronary flow. Percutaneous coronary intervention with stenting or coronary artery bypass graft operation should be considered for patients with ongoing ischemia, clinically unstable or spontaneous coronary artery dissection involving the left main or multiple proximal coronary dissections. Favorable prognosis has been reported in patients managed conservatively since resolution and healing can occur over time. The present case is the first documenting spontaneous coronary artery dissection and healing by high resolution imaging OCT.
